# Enhanced clindamycin delivery using chitosan-coated niosomes to prevent *Toxoplasma gondii* strain VEG in pregnant mice: an experimental study

**DOI:** 10.1186/s41182-024-00636-x

**Published:** 2024-09-29

**Authors:** Mitra Sadeghi, Seyed Abdollah Hosseini, Shahabeddin Sarvi, Pedram Ebrahimnejad, Hossein Asgaryan Omran, Zohre Zare, Shirzad Gholami, Alireza Khalilian, Seyedeh Melika Ahmadi, Fatemeh Hajizadeh, Mostafa Tork, Ahmad Daryani, Sargis A. Aghayan

**Affiliations:** 1 Toxoplasmosis Research Center, Communicable Diseases Institute, Mazandaran University of Medical Sciences, Sari, Iran; 2 Student Research Committee, Mazandaran University of Medical Sciences, Sari, Iran; 3 Department of Parasitology and Mycology, Faculty of Medicine, Mazandaran University of Medical Science, Sari, PC 48168-95475 Iran; 4 Pharmaceutical Sciences Research Center, Hemoglobinopathy Institute, Mazandaran University of Medical Sciences, Sari, Iran; 5 Department of Pharmaceutics, Faculty of Pharmacy, Mazandaran University of Medical Sciences, Sari, Iran; 6 Department of Immunology, Faculty of Medicine, Mazandaran University of Medical Science, Sari, Iran; 7 Department of Anatomical Sciences, Molecular and Cell Biology Research Center, Faculty of Medicine, Mazandaran University of Medical Sciences, Sari, Iran; 8 Biostatistics Department, Mazandaran University of Medical Sciences, Sari, Iran; 9 Laboratory of Molecular Parasitology, Scientific Center of Zoology and Hydroecology, NASRA, 7P. Sevak St., 0014 Yerevan, Armenia

**Keywords:** *Toxoplasma gondii*, VEG strain, Congenital, Clindamycin, Niosomes-coated chitosan

## Abstract

**Background:**

Congenital toxoplasmosis occurs when a pregnant woman becomes infected with *Toxoplasma gondii* (*T. gondii*) for the first time. Treatment typically involves antimicrobial medications, with spiramycin commonly used to prevent transmission. However, spiramycin's effectiveness is limited due to poor placental penetration. Clindamycin, another antibiotic, can cross the placenta but reaches the fetus at only half the maternal concentration. Encapsulating the drug in chitosan-coated niosomes (Cs-Nio) could enhance its effectiveness by targeting specific organs and ensuring sustained release. To address the challenges of using clindamycin, a niosome-coated chitosan system was investigated for treating congenital toxoplasmosis caused by the VEG strain of *T. gondii* in an animal model.

**Methods:**

Pregnant mice were infected with VEG strain of *T. gondii* on the 12th day of pregnancy, followed by treatment with various drugs across six groups. The treatments included chitosan-coated niosomes loaded clindamycin (Cs-Nio-Cli) and other controls. Parasitological evaluations (microscopic examination and real-time PCR), along with histopathological and immunological assessments were conducted to assess treatment efficacy. Finally, statistical analysis was conducted using GraphPad Prism 8.0 and SPSS 26, comparing test and control groups with *T* test and Mann–Whitney test. A *p* ≤ 0.05 was considered statistically significant.

**Results:**

The study found that treatment with Cs-Nio-Cli significantly reduced the number of *T. gondii* cysts in the brain and eyes (97.59% and 92.68%, respectively) compared to the negative control group. It also mitigated inflammatory changes, prevented cell death, and reduced vascular cuffs in the brain. In addition, Cs-Nio-Cli treatment decreased bleeding, placental thrombosis, and inflammatory cell infiltration in the placenta while improving eye tissue health by reducing retinal folds and bleeds. Immunologically, nanoclindamycin treatment resulted in lower TNF-α cytokine levels and higher IL-10 levels, indicating an enhanced anti-inflammatory response.

**Conclusions:**

Although Cs-Nio-Cli demonstrates promise in reducing the transmission of congenital toxoplasmosis and mitigating the effects of congenital toxoplasmosis, additional research is necessary to determine the optimal treatment regimens for the complete eradication of the parasite in the fetus.

**Supplementary Information:**

The online version contains supplementary material available at 10.1186/s41182-024-00636-x.

## Background

Toxoplasmosis, caused by the obligate apicomplexan protozoan *Toxoplasma gondii* (*T. gondii*), is a parasitic disease that can invade nucleated cells in warm-blooded hosts and occasionally in cold-blooded hosts [1]. In humans, toxoplasmosis is a significant opportunistic infection, particularly in immunocompromised individuals who may suffer from retinochoroiditis and fatal encephalitis if untreated [2]. In addition, *T. gondii* infection during pregnancy can have severe consequences for the embryo, leading to congenital toxoplasmosis [1, 3]. This condition occurs if a woman acquires *T. gondii* for the first time while pregnant, with the incidence of congenital infection in infants born to mothers who sero-converted during pregnancy ranging from 20% to 58% [4]. Congenital infection can result in fetal disorders such as hydrocephalus, microcephaly, retinochoroiditis, deafness, blindness, or even fetal death [5].

Despite the longstanding practice of screening pregnant women for *T. gondii* infection and providing prenatal treatment, the effectiveness of current therapies remains debated [6]. The standard treatment for toxoplasmosis in pregnant women involves administering spiramycin to reduce the risk of fetal transmission. Spiramycin achieves high concentrations in placental tissue, thereby lowering transmission risk. However, it has limitations in effectively clearing the parasite and penetrating fetal tissues. Prenatal administration can reduce transmission risk by up to 60% [7]. Consequently, researchers are investigating alternative medications, such as clindamycin and azithromycin, as potential substitutes in these scenarios.

Clindamycin, an antibiotic from the lincosamide group, crosses the placenta during pregnancy but reaches umbilical cord serum at approximately half the maternal concentration [8]. Despite its effectiveness, the absorption rate of clindamycin is about 50%. The physiological changes during pregnancy and childbirth can alter the pharmacokinetics of the drug, potentially impacting its therapeutic efficacy [9]. Consequently, we hypothesize that encapsulating the drug in a biodegradable system could significantly enhance its effectiveness while preserving its therapeutic properties.

Niosomes (Nio) are an advanced drug delivery system consisting of nanometer-scale vesicles made from non-ionic surfactants and cholesterol. They enhance drug penetration, improving efficacy and minimizing side effects by selectively targeting organs and ensuring controlled, gradual drug release. This targeted delivery is especially beneficial for treating infections such as toxoplasmosis, where effective drug penetration into placental and fetal tissues is essential [10].

Chitosan (Cs), a biodegradable and non-toxic polysaccharide, has become an essential material for nanoparticle preparation. Over the past two decades, chitosan and its derivatives have been widely utilized in medicine for their ability to prolong drug shelf life and improve drug absorption [11, 12]. In addition, chitosan’s anti-parasitic properties make it a promising candidate for the development of advanced drug delivery systems [13, 14].

In light of the limitations of existing treatments, this study seeks to address the challenges associated with clindamycin therapy by evaluating the potential of a niosome-coated chitosan (Cs-Nio) delivery system. Specifically, we investigated the efficacy of this system in treating congenital toxoplasmosis caused by the VEG strain (type III) of *Toxoplasma gondii* in a mouse model. Our objective is to enhance the therapeutic effectiveness of clindamycin, improve drug delivery to target tissues, and ultimately reduce the incidence and severity of congenital toxoplasmosis.

## Methods

### *T. gondii* strain

Cysts of the VEG strain of *Toxoplasma gondii* were procured from the Toxoplasmosis Research Center (TRC) at Mazandaran University of Medical Sciences. To reestablish parasite virulence, the strains were cultured via intraperitoneal injection in BALB/c mice. After 2 months, the brains of the infected mice were isolated, homogenized in PBS, and utilized for subsequent injections.

### Materials

Sorbitan monostearate (Span 60), polyethylene glycol sorbitan monostearate (Tween 60) (purity ≥ 99%) were purchased from Merck (Germany), while chitosan (Cs) (low molecular weight, purity ≥ 99%), acetic acid, tripolyphosphate, ethanol, and cholesterol (purity ≥ 99.7%, MW = 386.65) were obtained from Sigma-Aldrich Co. (Schnelldorf, Germany). The other chemicals and solvents used in this work were of the highest grade commercially available.

*Spiramycin drug:* Spiramycin, at a concentration of 3 M.I.U, was utilized in tablet form, supplied by Pharaonia Pharmaceuticals (analytical standard code: J01FA02). The tablet was dissolved in water and administered orally to the mice at a daily dosage of 400 mg/kg body weight.

*Clindamycin drug*: In this study, two formulations of clindamycin (Cli) were employed. The first was pure clindamycin, and the second was clindamycin encapsulated in chitosan-coated niosomes (Cs-Nio-Cli). Clindamycin hydrochloride hydrate, supplied in powdered form by Alborz Company in Tehran, Iran, was administered orally via gavage at a daily dosage of 200 mg/kg body weight.

### Manufacturing of Niosomes loaded clindamycin

To prepare the vesicular structure of niosomes, a precise combination of non-ionic surfactants (Span 60 and Tween 60), cholesterol (200–300 mg), and clindamycin (200–400 mg) with different HLB values (10–12) were used. This mixture was dissolved in 10 mL of chloroform and 5 mL of methanol to form the lipid phase. The solution was then evaporated using a rotary flash evaporator (LABOROTA 4000-efficient, Heidolph, Schwabach, Germany) at a controlled temperature of 60 °C for 30 min, resulting in a dry thin film. Subsequently, 30 mL of water was added to the flask to rehydrate the dry film, and the mixture was incubated at 60 °C for 1 h. To ensure a uniform and homogeneous dispersion of the niosome formulation, the mixture was processed using a small laboratory homogenizer (Hielscher Ultrasonics UP50H, Teltow, Germany). Sonication was performed at room temperature for 10 min using a 20% amplitude with a probe sonicator (Sonopuls mini-20, Bandelin Electronic type 3100, Berlin, Germany). Sonication played a critical role in forming stable niosome dispersions by promoting the effective dispersion of the niosome components. The various niosome formulations utilized in this study are presented in Table 1, each precisely engineered using specific ratios of nonionic surfactants, cholesterol, and drug to optimize niosome properties and drug encapsulation [15].

**Table 1:** Different niosomal formulations prepared for clindamycin encapsulation

Formulation	HLB	Cholesterol (mg)	Drug (mg)	Size (nm)	PDI (nm)	Zeta potential (mV)
F 1	10	200	200	108.5	0.297	−12.6
F 2	10	300	300	119.96	0.246	−9.93
F 3	10	200	400	116.16	0.263	−9.89
F 4	11	200	200	101.2	0.232	−9.71
F 5	11	300	300	148	0.238	−10.63
F 6	11	200	400	156.7	0.235	−9.20
F 7	12	200	200	131.66	0.278	−8.53
F 8	12	300	300	161.66	0.228	−9.76
F 9	12	200	400	152.4	0.340	−8.72

### Determination of percent encapsulation

The drug encapsulation percentage was quantified utilizing UV–Vis's spectrophotometry (Nanodrop, Thermo Fisher Scientific, Waltham, MA, USA). The niosomal liquid dispersion was analyzed at a wavelength of 218 nm, specific to clindamycin. Absorbance values obtained were employed to calculate the encapsulation percentage based on a pre-established standard curve.

### Preparation of chitosan-coated niosomes

At ambient temperature, a 25 mL chitosan solution (0.05 g in 0.01% acetic acid) was mixed with an aqueous solution of clindamycin-loaded niosomes (Nio-Cli) on a magnetic stirrer. The mixture was stirred, and excess acetic acid was removed by centrifugation. Subsequently, 10 mL of 10 mg/mL triphosphate was added dropwise until a suspension of chitosan-coated clindamycin-loaded niosomes (Cs-Nio-Cli) was formed. The suspension was then freeze-dried for 24 h, resulting in a white residue, which was stored at −20 °C for future use.

### Characterization of nanoparticle formulations

#### Nanoparticle measurement

Determination of the hydrodynamic particle size, polydispersity index (PDI) and zeta potential of chitosan-coated niosomes loaded with clindamycin was performed using Zetasizer (Nano ZS90, Malvern Instruments, UK) according to Manca et al. [16].

#### Determining the morphology of nanoparticles by SEM

The surface characteristics and morphology of the nanoparticles were analyzed using a scanning electron microscope (SEM, JOEL, JSM-5300, Japan) [17]. Prior to SEM examination, the samples were lyophilized and coated with platinum.

### Mating and infection

In a controlled setting, female mice were paired with males, and the onset of pregnancy was confirmed by observing weight gain and the presence of a vaginal plug. Subsequently, males were separated, and groups of three females were co-housed until the time of infection [18]. On the 12th day of gestation, the mice were orally administered 20 cysts of the *T. gondii* VEG strain suspended in PBS, in preparation for subsequent analysis.

### Experimental design

In this study, 36 pregnant mice were categorized into six groups. The groups were as follows: GI: uninfected and untreated (normal group), GII: untreated infection (negative control), GIII: infected and treated with clindamycin (200 mg/kg), GIV: infected and treated with spiramycin (400 mg/kg) (positive control), GV: infected and treated with Cs-Nio-Cli (200 mg/kg), GVI: infected and treated with Cs-Nio (30 mg/kg). Groups III to VI received oral drug administration from the time of parasite inoculation until delivery, with the drugs diluted in 0.5 ml of PBS. The drug doses were adjusted according to the recommended doses for pregnant women.

### Evaluation of the drug efficacy

#### Tissue sampling

Two days before parturition, placentas were surgically extracted from pregnant mice via cesarean section. Six week postpartum, the offspring were humanely euthanized using a ketamine overdose to collect brain and eye tissues. These tissues were then divided into left and right sections. The left halves were homogenized, and a 10 µl sample was microscopically examined to assess parasite load. The remaining homogenized tissue from the left halves was stored at −20 °C for further evaluation using real-time PCR. The right halves were preserved in 10% formalin for subsequent histopathological examination.

#### Counting the *T. gondii* tissue cysts number

To evaluate the drug’s effects, we counted the number of cysts present in 0.01 grams of mice brain and eye tissues using microscopic examination (40× magnification, NIKON, Japan) and Giemsa staining (MERCK, Germany) [19].

#### Parasite burden by real-time PCR

Genomic DNA was extracted from tissues, including the brains and eyes of newborn mice and the placentas of pregnant mice, using the DENAzist Animal Tissue DNA Isolation Kit (Iran). The B1 gene was then amplified using AB Applied Biosystems as an initial step in real-time PCR, following established protocols to determine the *T. gondii* load [20]. The parasite burden of *T. gondii* was assessed by comparing threshold cycle (CT) values with standard curves derived from serially diluted RH strain DNA. Distilled water was used as the negative control, while DNA from RH strain tachyzoites served as the positive control.

#### Histopathological study

Tissue specimens from each group were collected individually and fixed in neutral buffered formalin for 48 h. After paraffin embedding, 5-μm-thick sections were prepared using a rotary microtome (Leica Model RM 2145, Germany) and stained with hematoxylin and eosin (H&E). The slides were then examined for histopathological alterations under light microscopy (NIKON, Japan) [21].

#### Immunological study and cytokines assay

To evaluate cytokine levels, blood samples were obtained from the hearts of mice 6 week postpartum using sterile vacuum tubes containing EDTA. Serum concentrations of TNF-α and IL-10 were measured using an ELISA kit (Carmania Parsgen, Iran) in accordance with the manufacturer’s instructions. The results were expressed in pg/mL [14].

### Statistical analysis of the data

The collected data were analyzed statistically using GraphPad Prism software version 8.0 (GraphPad Software, Inc., San Diego, CA, USA) and SPSS software version 26. Differences between the test groups (both positive and negative) and control groups were evaluated using the *T* test and Mann–Whitney test. A *p* value of 0.05 or lower was considered statistically significant in this study.

## Results

### Niosomes particle size measurement and zeta potential

The niosomal formulation was subjected to thorough characterization to assess its physical properties and stability. Among the different formulations tested, formulation F4 exhibited the smallest size (101.2 nm), indicating successful formulation optimization (Supplementary file 1). The measured zeta potential for this formulation was −9.71 mV. Typically, zeta potential values farther from zero in either direction suggest increased colloidal stability due to stronger electrostatic repulsion between particles, which prevents aggregation (Supplementary file 2). The Polydispersity Index (PDI) is another important parameter used to assess the uniformity of nanoparticles in a formulation. It quantifies the distribution of particle sizes within a sample. In addition, a PDI value of 0 indicates a uniform size distribution, while values closer to 1 suggest a broader distribution of sizes. In the context of the niosomal formulation mentioned, a PDI value of 0.232 indicates a relatively narrow size distribution. This suggests that the nanoparticles within the formulation are fairly uniform in size, which is advantageous for consistent behavior in biological systems and predictable release profiles (Table 1).

Table 1 provides a comprehensive overview of the mean size, zeta potential, and polydispersity index of the different niosomal formulations evaluated in the study. The table presents information regarding the physical characteristics of each formulation, aiding in the selection of the optimized formulation for further investigation. The precise characterization of the niosomal formulation ensures a thorough understanding of its physicochemical properties and aids in assessing its potential for drug delivery and therapeutic applications.

### Scanning electron microscopy (SEM)

**Fig. 1:**
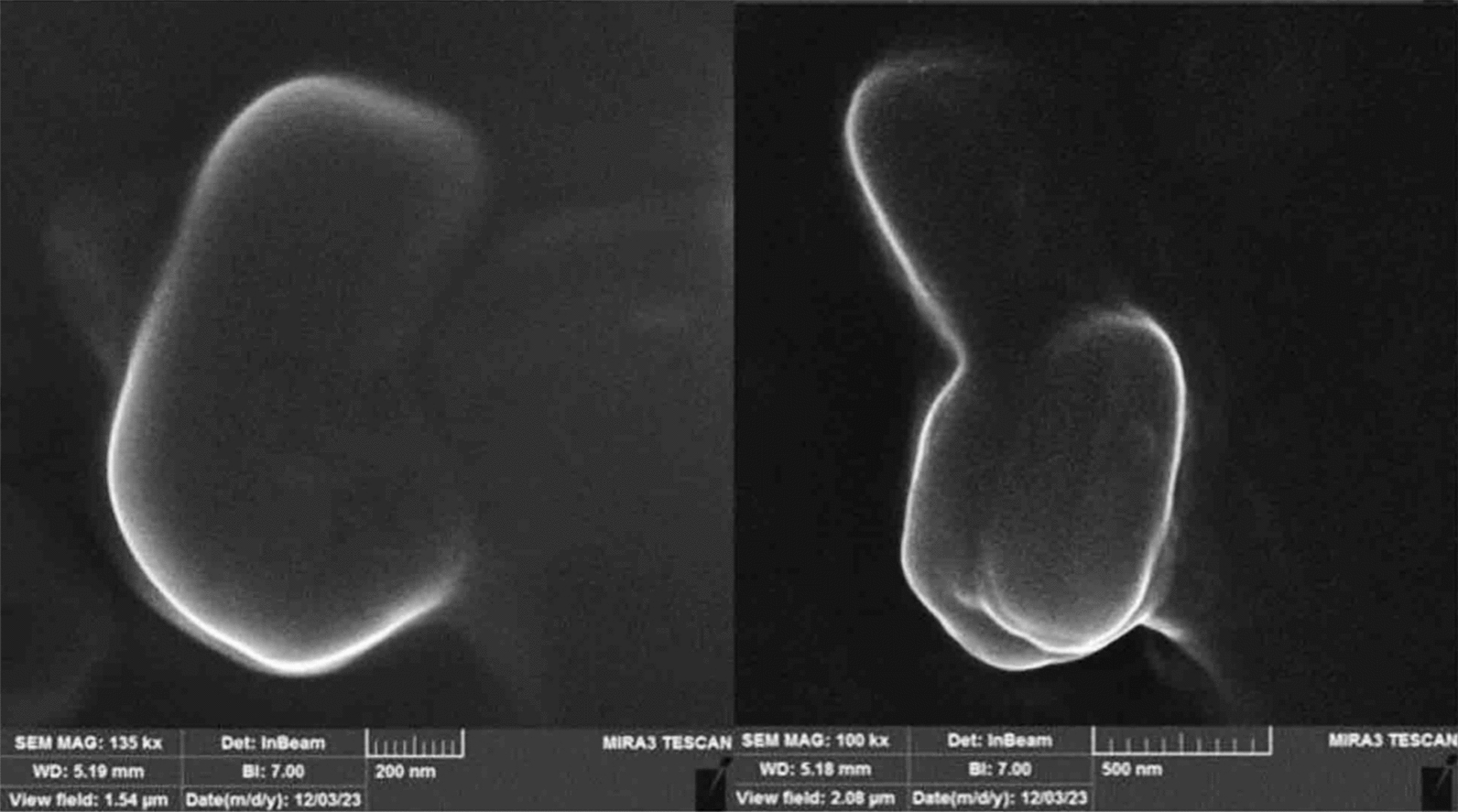
Scanning electron microscopy (SEM) of Cs-Nio-Cli

The morphological characterization of the optimized niosomal formulation was conducted using scanning electron microscopy (SEM). The SEM images revealed that the niosomes exhibited a spherical shape with a uniform size distribution. In addition, the images demonstrated that the niosomes were well-segregated, indicating their individual presence within the formulation. SEM analysis also facilitated the assessment of the surface characteristics of the optimized formulation, revealing that the nanoniosomes possessed a smooth surface and internal porosity. This is crucial for ensuring proper interaction and compatibility of the niosomes with the target site (Fig. 1).

### Examining the amount of drug loading in nanoparticle

The drug loading efficiency and final concentration were assessed by quantifying the free clindamycin on the nanoparticle surface post-centrifugation using high-pressure liquid chromatography (HPLC). In this study, the initial drug amount was 200 mg, with 95.31 mg successfully encapsulated within the Niosomes.

### Microscopic determination of *Toxoplasma* parasite load

**Fig. 2:**
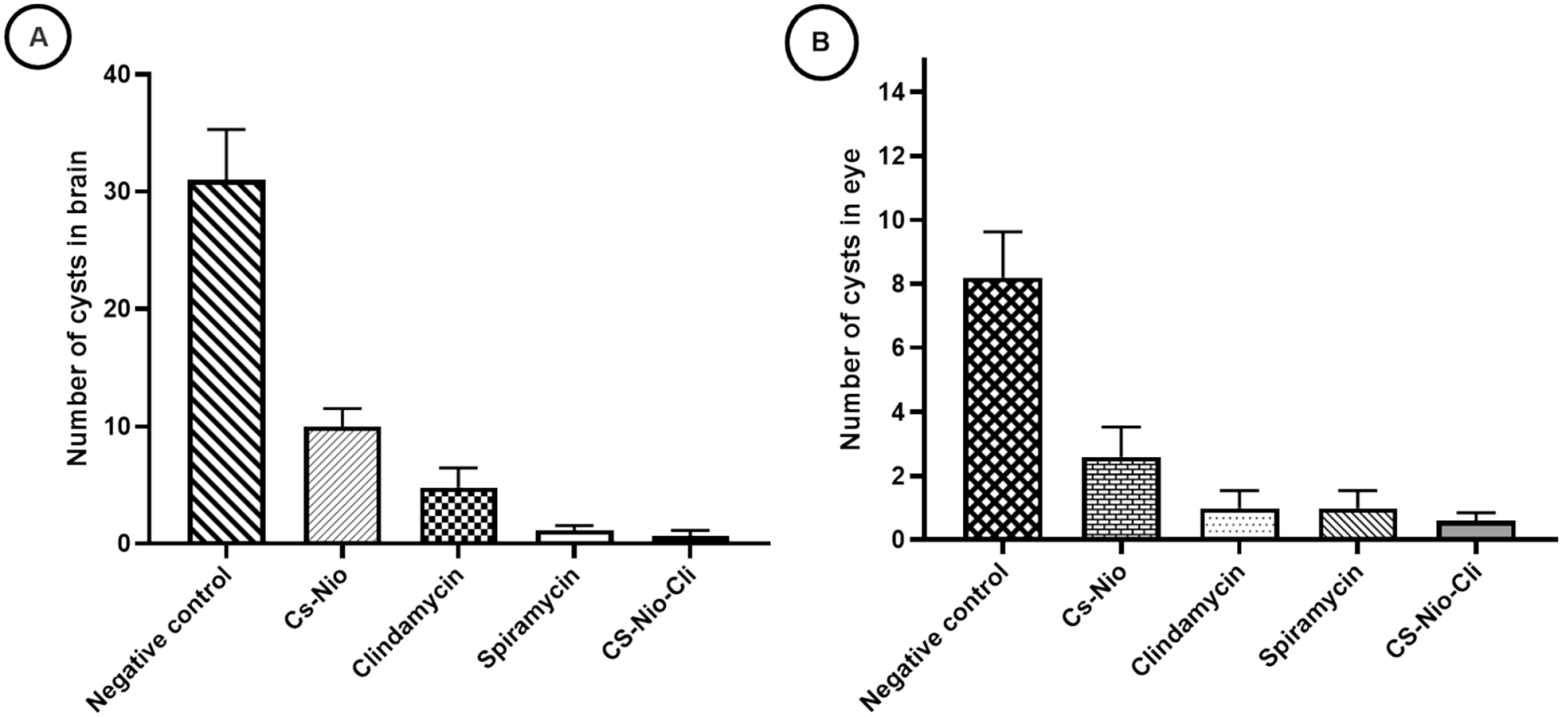
*Toxoplasma* cyst burden per hippocampus brain section and eye in various infected groups. Data represent the mean ± standard error of the mean (SEM)

As shown in Fig. 2, the mean reduction in brain and eye cysts was observed in all treated groups compared to the negative control group, and the difference was statistically significant. In the groups treated with Cs-Nio-Cli and spiramycin, there was a significant reduction in brain cysts (97.59% and 96.99%, respectively) and eye cysts (92.68% and 87.80%, respectively) compared to the untreated negative control group. So that the lowest number of brain and eye cysts was observed in the Cs-Nio-Cli group with a statistically significant difference compared to the negative control. This reduction was moderate in the group treated with clindamycin (*P* < 0.05). While the number of cysts observed in the brain and eye tissue in the group treated with Cs-Nio-Cli was lower compared to the control positive (spiramycin) group, however, no statistically significant differences were observed between those two groups (*P* > 0.05).

### Estimation of the parasite burden by quantitative real time PCR

The successful identification of the B1 gene of *T. gondii* was achieved through QPCR utilizing a 98 bp amplification fragment. As shown in Table 2, in samples collected from mice treated, the expression levels of the B1 gene exhibited a significant reduction compared to the negative control group (*P* < 0.05). Nevertheless, this decreases in the spiramycin and Cs-Nio-Cli groups significantly surpassed that observed in the clindamycin-treated group.

**Table 2:** Real-time PCR analysis of *T. gondii* parasite burden and CT values in brain, eye, and placenta tissues of different groups

Groups	CT (Mean ± SD of parasite load)								
	Brain	Statistical significance (*P* < 0.05)		Eye	Statistical significance (*P* < 0.05)		Placenta	Statistical significance (*P* < 0.05)	
		Negative control	Positive control		Negative control	Positive control		Negative control	Positive control
PBS (negative control)	25.21 ± 0.79 (2700 ± 0.94)	Ref*	0.000	25.91 ± 0.91 (2700 ± 1200)	Ref	0.01	26 ± 0.78 (2700 ± 400)	Ref	0.01
Cs- Nio	27.14 ± 0.17 (1153.64 ± 102.64)	0.000	0.004	27.42 ± 0.29 (960.38 ± 131.80)	0.15	0.003	26.27 ± 0.57 (2181.8 ± 595.8)	0.73	0.008
Clindamycin	27.45 ± 0.24 (930.2 ± 67.19)	0.009	0.002	29.11 ± 0.34 (300.5 ± 49.56)	0.04	0.009	29.11 ± 0.24 (300.68 ± 49.66)	0.03	0.02
Spiramycin (positive group)	33 ± 0.95 (29.62 ± 12.63)	0.000	Ref	31.79 ± 0.13 (46.95 ± 30.2)	0.01	Ref	30.75 ± 0.07 (100.96 ± 9.08)	0.01	Ref
Cs-Nio-Cli	32.34 ± 0.57 (33.28 ± 8.98)	0.002	0.45	31.01 ± 0.89 (88 ± 35.92)	0.03	0.34	30.75 ± 0.35 (99.52 ± 14.52)	0.01	0.98
Normal group	36.84 ± 0.73	0.000	0.005	36.84 ± 0.73	0.001	0.003	36.84 ± 0.73	0.001	0.002

^*^Ref: In each statistical column, one of the positive control or negative control groups is considered as the reference group for comparison

In particular, the lowest parasite load was observed in brain, eye and placenta samples with a very small difference in Cs-Nio-Cli and spiramycin-treated mice. While the group treated with nano-clindamycin showed a significant decrease in gene expression and parasite load compared to the negative control group. However, when compared to the positive control group (spiramycin), it showed almost the same level of success with spiramycin and statistically significant difference was not observed (*P* > 0.05).

### Histopathological evaluation

**Fig. 3:**
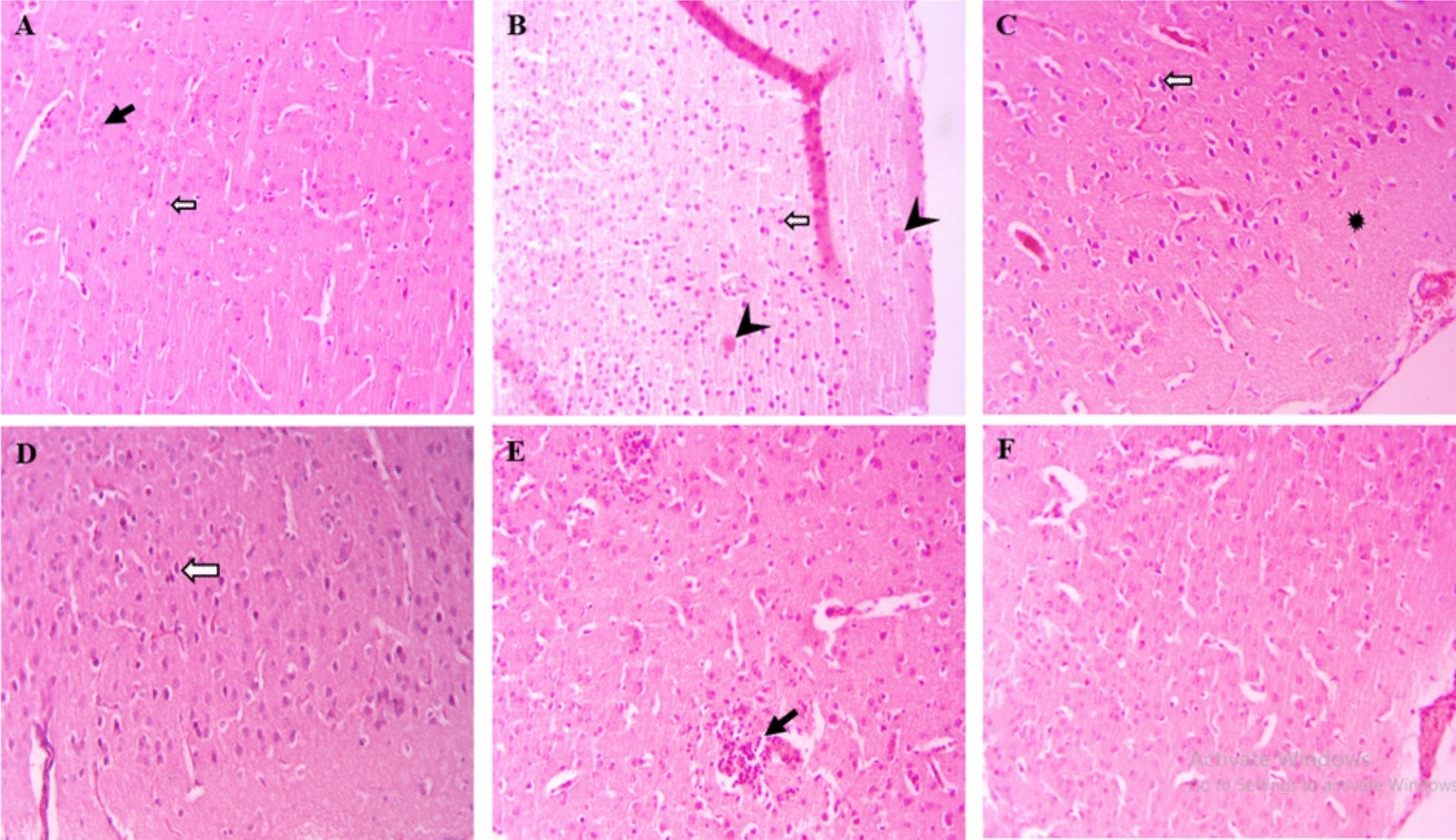
Microscopic image of mouse brain tissue, H&E staining, image magnification × 200. **A** In the control group, normal brain tissue including neurons (black arrow) and normal glial cells (white arrow) can be seen. **B** In histology sections of brain tissue in groups infected with VEG strain without drug, **C** infected treated with chitosan-coated niosomes without drug, **D** infected treated with spiramycin, **E** infected treated with clindamycin, **F** infected treated with Cs-Nio-Cli, inflammatory cell infiltration with glial cell proliferation (black arrow), vacuolar neuropil (star), wrinkled and intensely stained neurons (white arrow) and perivascular cuff (head arrow) are shown

**Fig. 4:**
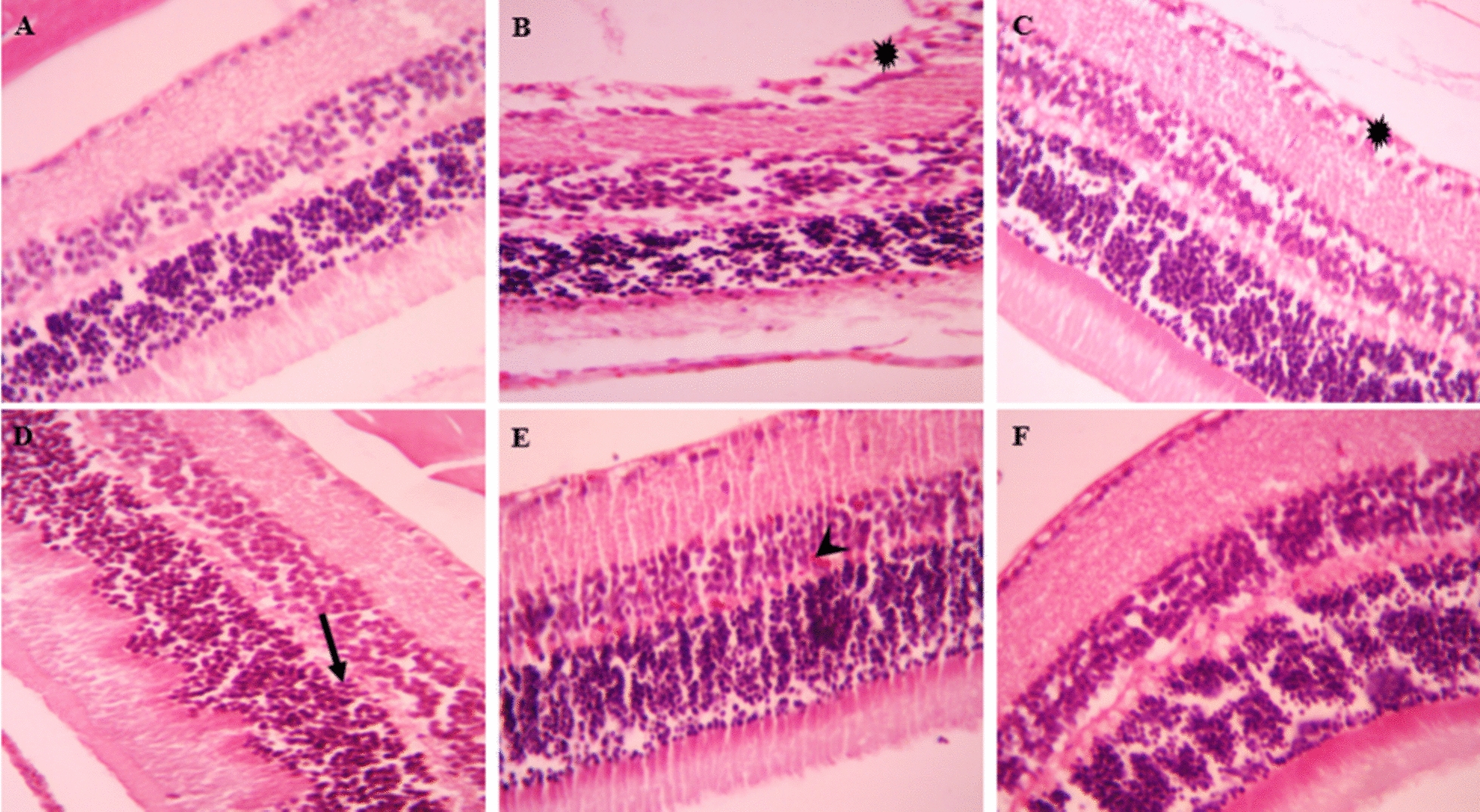
Microscopic image of mouse eye tissue, H&E staining, image magnification × 400. **A** In the control group, the normal tissue of different layers of the eye can be seen. **B** In eye tissue histology sections in groups infected with VEG strain without drug, **C** infected treated with chitosan-coated niosomes without drug, **D** infected treated with spiramycin, **E** infected treated with clindamycin, **F** infected treated with Cs-Nio-Cli, retinal folding and destruction of its structure (black arrow), cellular edema and vacuolization (star) and retinal micro hemorrhage (head arrow) are shown

**Fig. 5:**
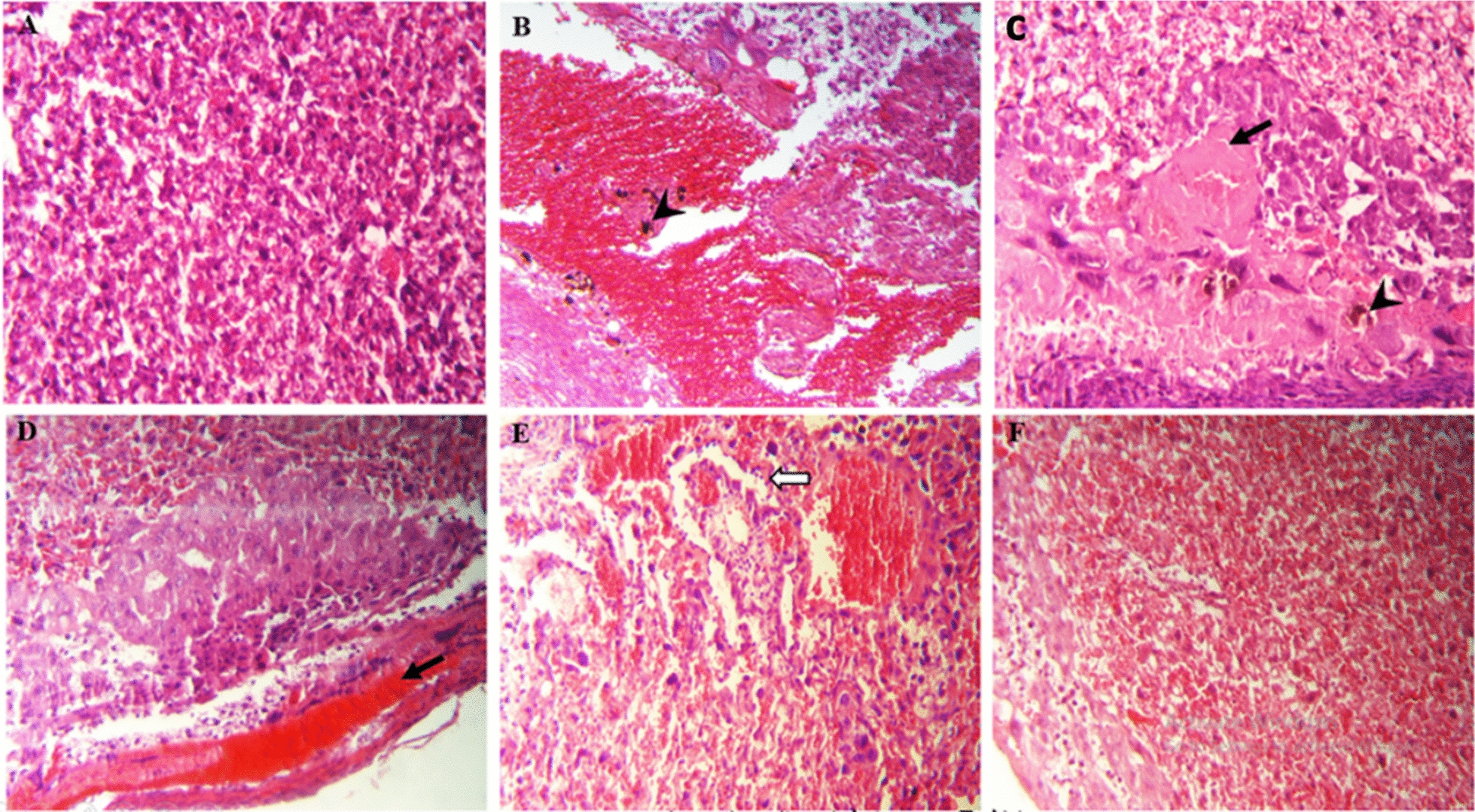
Microscopic image of placenta tissue in mice, H&E staining, image magnification × 200. **A** Control group, the normal tissue of placental labyrinth layer is seen. **B** In histology sections of placenta tissue in groups infected with VEG strain without drug, **C** infected treated with chitosan-coated niosomes without drug, **D** infected treated with spiramycin, **E** infected treated with clindamycin, **F** infected treated with Cs-Nio-Cli, thrombosis and hemorrhage (black arrow), accumulation of inflammatory cells (white arrow) and local calcification (head arrow) are shown

Histopathological changes of the brain, eye and placenta tissues were investigated among all groups, showing a difference in the amount of tissue damage compared to the untreated control group. As shown in Figs. 3, 4, and 5, no changes in histological damage were observed in the brain, eye, and placenta tissues of the healthy control group. In contrast, in the untreated infected mice and the infected group treated with Cs-Nio, histological damage was noted, including cell infiltration inflammation, proliferation of glial cells, perivascular cuff, and diffuse neurodegeneration in the brain, as well as retinal folds, structural destruction, retinal microhemorrhages, edema, and cell vacuolization in the eye. However, the cuff around cerebral vessels and the Rosette-like foci structures were not observed in the eyes of the Cs-Nio group compared to the untreated group.

In addition, there were tissue injuries such as hemorrhage, placental thrombosis, and accumulation of inflammatory cells in the placental labyrinth and decidua, as well as localized calcification in the placental labyrinth.

Administration of clindamycin, spiramycin, and Cs-Nio-Cli led to decreased histological damage in brain, eye, and placental tissues compared to the untreated infected group. Notably, the groups receiving spiramycin and Cs-Nio-Cli exhibited significant improvements in histopathological changes.

### Immunological study

**Fig. 6:**
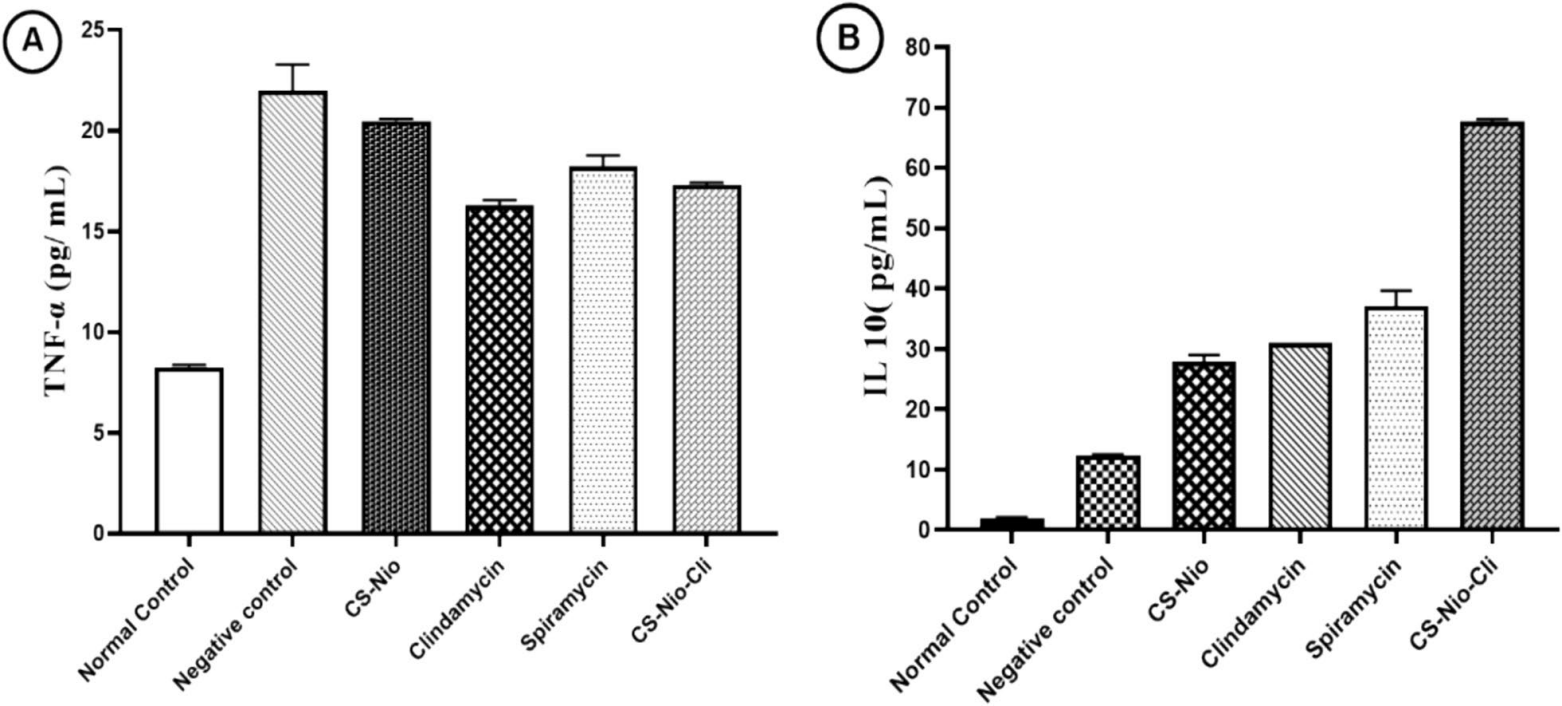
Serum level of IL-10 and TNF-α of different groups. Data represent the mean ± SEM

Serum levels of IL-10 and TNF-α cytokines were measured in the blood of all groups of mice. As shown in Fig. 6, healthy mice (control group) had the lowest levels of TNF-α and IL-10 cytokines in their blood, respectively (8.21 ± 0.19 and 2 ± 0.1). While untreated infected mice (negative control), had the strongest response and showed the highest levels of TNF-α cytokine (21.99 ± 2.21). Although, the production of cytokine TNF-α was almost the same between mice treated with spiramycin (positive control) and mice treated with Cs-Nio-Cli, and no statistically significant difference was observed between the two groups (*P* > 0.05). However, the mice treated with Cs-Nio-Cli compared to the infected positive control group (spiramycin) showed higher levels of IL10 than other treated groups, and there was a statistically significant difference between the two groups of spiramycin and Cs-Nio-Cli (*P* < 0.05). In addition, mice treated with clindamycin induced moderate levels of secretion of both cytokines compared to the negative control and positive control (spiramycin) groups.

## Discussion

*Toxoplasma gondii* (*T. gondii*) is the cause of toxoplasmosis that multiplies only in living cells. The human infection may be acquired by ingestion of oocysts excreted by cats and contaminating soil or water, or by eating tissue cysts that remain viable in undercooked meat of infected animals and it may generate devastating damage in newborns, immunocompromised patients and fetuses [1]. Mother-to-child transmission of the parasite occurs only when infection is acquired for the first-time during pregnancy. Congenital toxoplasmosis (CT) may present as a mild or severe neonatal disease. The stage of pregnancy during which maternal exposure occurs significantly impacts the likelihood of embryo or fetus infection and the clinical outcome in humans. If the mother contracts an acute infection, *T. gondii* can spread through the placenta via the bloodstream, reaching fetal circulation. Furthermore, if the infection occurs close to the period of embryogenesis, the resulting clinical impact tends to be more severe [22]. Consequently, the risk of severe abnormalities is higher when the fetus is infected during the first or second trimester of pregnancy. Nonetheless, early treatment of the fetus remains essential to minimize the risk of severe abnormalities [22].

In pregnancy, treating toxoplasmosis involves balancing infection management with fetal protection. While spiramycin is commonly used to reduce the risk of transmitting the infection to the unborn child [23]. Its limited ability to cross the placenta results in only a 60% reduction in congenital toxoplasmosis transmission [7]. Therefore, alternative drugs like clindamycin are being explored as potential option.

Currently, Clindamycin is extensively utilized as an alternative treatment for toxoplasmosis, either as a monotherapy or in combination with pyrimethamine [24]. Numerous in vitro studies have demonstrated clindamycin’s inhibitory effects on *T. gondii*, highlighting its capacity to disrupt parasite growth and reproduction. In addition, the combination of clindamycin and pyrimethamine has shown efficacy in managing toxoplasmic encephalitis in immunocompromised patients, particularly when standard treatments are poorly tolerated or ineffective [25]. Clindamycin effectively crosses the placenta, achieving umbilical cord serum levels approximately half that of maternal levels [8]. Notably, evidence robustly supports the safe use of topical or oral clindamycin during pregnancy, with no reported teratogenic effects [26].

Clindamycin has shown promising results in animal models, particularly in mice, by reducing *T. gondii* parasite levels and alleviating toxoplasmosis symptoms. However, data on its optimal dosage for treating congenital infections remain limited [27]. A systematic review and meta-analysis demonstrated that clindamycin used before 22 completed weeks of gestation in women with objective evidence of abnormal genital tract flora can significantly reduce the rate of late miscarriage and early preterm birth [28]. In addition, McGreedy et al. (2001) found that clindamycin is considered safe during the first trimester, with no increase in birth defects among 65 infants whose mothers were treated with clindamycin and quinine for malaria later in pregnancy [29].

While approximately half of the administered clindamycin is absorbed, pregnancy and childbirth can further complicate its pharmacokinetics [9]. To address these challenges, we propose a novel strategy: encapsulating clindamycin within a biodegradable delivery system.

Our hypothesis is that nanoparticles, known for their ability to improve drug bioavailability and reduce toxicity, can effectively deliver clindamycin. We focus on two promising nanoparticle systems: chitosan nanoparticles and niosomes.

This study investigated the potential of a novel nano-carrier system, chitosan-coated niosomes, to enhance clindamycin delivery and explore its impact on the vertical transmission of *T. gondii* VEG strain (type III) in a mouse model. Chitosan-coated niosomes (Cs-Nio-Cli) leverage the advantages of both niosomes and chitosan to improve drug encapsulation, stability, and targeted delivery, potentially overcoming the pharmacokinetic challenges posed by pregnancy and enhancing the therapeutic efficacy against congenital toxoplasmosis. In the present study, compared to the untreated control group, the treated groups had a significant effect on the rate of congenital infection caused by the VEG strain of *Toxoplasma* and were able to reduce the residual infection and the number of cysts in the brain and eye tissue. While Cs-Nio-Cli has been somewhat more successful in reducing the parasite load and reducing the severity of toxoplasmosis compared to other treated groups (Treat with spiramycin and clindamycin), so that the greatest reduction was in brain cysts (97.59%, 96.99% and 84.94%, respectively) and eye cysts (92.68%, 87.80% and 87.8%, respectively), but The difference in the number of brain and eye tissue cysts between the groups treated with clindamycin and Cs-Nio-Cli compared to the positive control group (spiramycin) was not significant. AL-Akash et al. [30] demonstrated significant outcomes in a mouse model of congenital toxoplasmosis using a combined Malarone and clindamycin regimen. All treated animals survived, and brain tissue cysts were completely eliminated, suggesting a potential cure [30]. In addition, the results of the present study are consistent with the study conducted by Hagras et al. [31]. In their study, the effects of spiramycin–metronidazole and spiramycin-loaded chitosan nanoparticles (CS) were tested in comparison to the current spiramycin treatment of *T. gondii* on tissue penetration and blood–brain barrier (BBB) crossing. The results showed that the maximum survival time of more than 200 days without mortality was observed on the sacrifice day (eighth) in mice receiving NPs with spiramycin. In addition, nanoparticles containing spiramycin showed the highest percentage of significant reduction of tachyzoites (about 90% reduction) in liver, spleen, and brain compared to other drugs used, which indicate successful BBB bypass. As a result, nanoparticles containing spiramycin showed the highest efficiency in the treatment of acute toxoplasmosis [31].

Quantitative PCR evaluation showed that *T. gondii* parasite load in brain tissue, eye and placenta in all groups of treated mice showed a significant decrease compared to the untreated control group, which indicates the effectiveness of these drugs (*P* < 0.05). However, Q-PCR analysis showed that both clindamycin and Cs-Nio-Cli treatments, similar to spiramycin, did not completely halt *T. gondii* embryo transmission. It is worth noting that there was no statistically significant difference in the reduction of parasite load in the brain, eye and placenta between the group treated with Cs-Nio-Cli compared to the positive control group (spiramycin) (*P* > 0.05). Interestingly, the significant increase in parasite load reduction was observed in clindamycin and Cs-Nio-Cli-treated groups and is consistent with the findings reported by Araujo et al. [32], which demonstrated that clindamycin effectively prevented congenital transmission during the acute infection in mother [32].

In the present study, treatment with clindamycin, like spiramycin, was able to reduce histological changes compared to the untreated control group. The significant improvement observed in inflammatory changes in subgroups treated with spiramycin and clindamycin is consistent with the results of the study by AL. Akash et al. It shows a significant improvement during the treatment with two drugs, malarone and clindamycin at a dose of 200 mg/kg, because the retina looked normal with some necrosis in the two layers of ganglion cells and disintegration in the inner and outer layers of the nucleus. In addition, the brain appeared normal and free of tissue cysts [30].

In the Cs-Nio-Cli-treated group, we observed a significant reduction in pathological damage compared to other groups. This reduction aligns with the TNF secretion levels seen in mice treated with nanoclindamycin. These findings are consistent with Etewa et al. [33] observations, where spiramycin-loaded chitosan nanoparticles (SLCNs) effectively mitigated pathological effects in the liver, spleen, eye, and brain of mice infected with *Toxoplasma* RH and Me49 strains. As a result, they show that the loading of spiramycin on chitosan nanoparticles increased its antiparasitic effect on acute and chronic *T. gondii* infection [33]. In accordance with our findings, Osama et al. [34] examined the impact of nanoparticles on enhancing the antiparasitic efficacy of spiramycin. They used spiramycin loaded on carboxymethyl chitosan nanoparticles (CMC-Np) to treat toxoplasmosis, evaluating the results through parasitological and histopathological methods. The study's results revealed a significant reduction in parasite load and an improvement in histopathological changes in the groups treated with spiramycin loaded on carboxymethyl chitosan nanoparticles compared to the other groups [34].

Inflammation plays an important role in disease processes and the involvement of inflammatory cytokines is significant. These cytokines can contribute to tissue damage [35]. In the present study, the average cytokine production of TNF-α in all groups was compared to the untreated infected group, and this result is in line with the findings of other studies that reported that TNF-α levels peak during acute *T. gondii* infection and it shows an important role for this cytokine in the pathogenesis of the acute phase of the disease [35]. In the present study, a decrease in the level of cytokine TNF-α was observed following treatment with Cs-Nio-Cli, which indicates an increase in the improvement of the cellular immune response. Furthermore, these findings align with GabAllah et al. study (2021), which demonstrated reduced tissue inflammation in the spiramycin-loaded gold nanoparticle-treated groups compared to both the spiramycin-treated group and the untreated group [36].

Based on this, it can be explained that Cs-Nio can act as an immune stimulator and this may be due to some of its properties such as anti-inflammatory and antimicrobial activities. Chitosan nanoparticles may play a significant role in this enhancement by inducing robust immune responses, particularly cellular immunity, as observed by Hamad et al. [37].

IL-10 plays a crucial role in balancing protective immunity and immunopathology. It counteracts the effects of TNF-α, blocking genes and functions normally stimulated by TNF-α [38].Therefore, the increase in IL-10 levels that we detected in the infected groups treated with Cs-Nio-Cli or spiramycin may contribute to the reduction of TNF-α production Our results showed not only a decrease in TNF levels but also a relative increase in IL10 production in the Cs-Nio-Cli group compared to spiramycin, which could be very important for maintaining pregnancy Similarly, in the study of Hamad et al*.* [37], TNF-α showed decreased levels after treatment with spiramycin plus chitosan compared to spiramycin alone, which improved the immune response. The latter could explain why chitosan was proposed as an immunostimulatory [37].

Overall, research indicates that clindamycin can inhibit the growth and reproduction of *T. gondii*, suggesting its potential utility in parasite control. Clinical studies suggest that clindamycin’s effectiveness against toxoplasmosis may be similar to its antibacterial action, which involves targeting the ribosomes within the parasite’s cytoplasm and mitochondria [39, 40].

Although spiramycin is usually the preferred first-line treatment for congenital toxoplasmosis, clindamycin can serve as a useful alternative or adjunctive therapy in case of resistance, intolerance, or limited access to the first-line drugs.

Early administration of spiramycin may prevent the transmission of infection to the fetus but is unlikely to halt existing brain infections, which represent the most severe consequence of congenital toxoplasmosis in humans. Similarly, in the present study, although clindamycin significantly reduced the burden of tissue cysts, it did not completely eliminate parasites from the tissue samples. This outcome is not unexpected, as clindamycin, a lincomycin antibiotic, has poorly characterized penetration into the nervous system and cerebrospinal fluid [41].

In this study, Cs-Nio-Cli demonstrated significantly greater efficacy than either spiramycin or clindamycin alone in reducing tissue cysts, particularly in the brain, and mitigating brain injuries. This enhanced efficacy is attributed to the niosome-coated chitosan’s ability to improve drug delivery and penetration, especially across the blood–brain barrier (BBB). The targeted delivery system ensures a higher concentration of clindamycin in brain tissues and promotes sustained release, thereby enhancing the overall therapeutic impact. Consequently, Cs-Nio-Cli may represent a promising treatment strategy for congenital toxoplasmosis, offering superior protection against the parasite by effectively crossing the BBB and targeting the central nervous system.

## Conclusion

This research underscores the potential of Cs-Nio-Cli as a component of a comprehensive treatment strategy aimed at reducing vertical transmission of *Toxoplasma*. While it does not achieve complete eradication of the parasite, challenges remain in ensuring effective drug penetration into the placenta and central nervous system, which are critical for preventing vertical transmission and safeguarding fetal development.

While Cs-Nio-Cli shows promise in reducing the transmission of congenital toxoplasmosis, further research is essential to determine the optimal treatment regimens for clindamycin in reducing vertical transmission and achieving complete eradication of the parasite in the fetus.

## Supplementary Information

Supplementary material 1.

Supplementary material 2.

## Data Availability

All data is provided in the manuscript and in additional files. All data are available on request.

## Abbreviations

Nio: Niosomes
CS: Chitosan
CS-Nio: Chitosan-coated niosomes
Cli: Clindamycin
CS-Nio-Cli: Chitosan-coated niosomes with clindamycin
