# Correction: Enhanced clindamycin delivery using chitosan-coated niosomes to prevent *Toxoplasma gondii* strain VEG in pregnant mice: an experimental study

**DOI:** 10.1186/s41182-024-00643-y

**Published:** 2024-10-15

**Authors:** Mitra Sadeghi, Seyed Abdollah Hosseini, Shahabeddin Sarvi, Pedram Ebrahimnejad, Hossein Asgaryan Omran, Zohre Zare, Shirzad Gholami, Alireza Khalilian, Seyedeh Melika Ahmadi, Fatemeh Hajizadeh, Mostafa Tork, Ahmad Daryani, Sargis A. Aghayan

**Affiliations:** 1 Toxoplasmosis Research Center, Communicable Diseases Institute, Mazandaran University of Medical Sciences, Sari, Iran; 2 Student Research Committee, Mazandaran University of Medical Sciences, Sari, Iran; 3 Department of Parasitology and Mycology, Faculty of Medicine, Mazandaran University of Medical Science, Sari, 48168-95475 PC Iran; 4 Pharmaceutical Sciences Research Center, Hemoglobinopathy Institute, Mazandaran University of Medical Sciences, Sari, Iran; 5 Department of Pharmaceutics, Faculty of Pharmacy, Mazandaran University of Medical Sciences, Sari, Iran; 6 Department of Immunology, Faculty of Medicine, Mazandaran University of Medical Science, Sari, Iran; 7 Department of Anatomical Sciences, Molecular and Cell Biology Research Center, Faculty of Medicine, Mazandaran University of Medical Sciences, Sari, Iran; 8 Biostatistics Department, Mazandaran University of Medical Sciences, Sari, Iran; 9 Laboratory of Molecular Parasitology, Scientific Center of Zoology and Hydroecology, NASRA, 7P. Sevak St., 0014 Yerevan, Armenia

**Correction: Tropical Medicine and Health (2024) 52:64** 10.1186/s41182-024-00636-x

Following publication of the original article [1], the authors identified an error in the author name of Pedram Ebrahimnejad.

The incorrect author name is: Pedram Ebrahim Nejad

The correct author name is: Pedram Ebrahimnejad

The author group has been updated above and the original article [1] has been corrected.
