# Supplementary figures and images for: Enhanced clindamycin delivery using chitosan-coated niosomes to prevent Toxoplasma gondii strain VEG in pregnant mice: an experimental study
Source: Trop Med Health. 2024 Sep 29;52:64. doi: 10.1186/s41182-024-00636-x (PMC11439218; doi:10.1186/s41182-024-00636-x)

**Supplementary file (S1)**. Size distribution by intensity (d. nm)


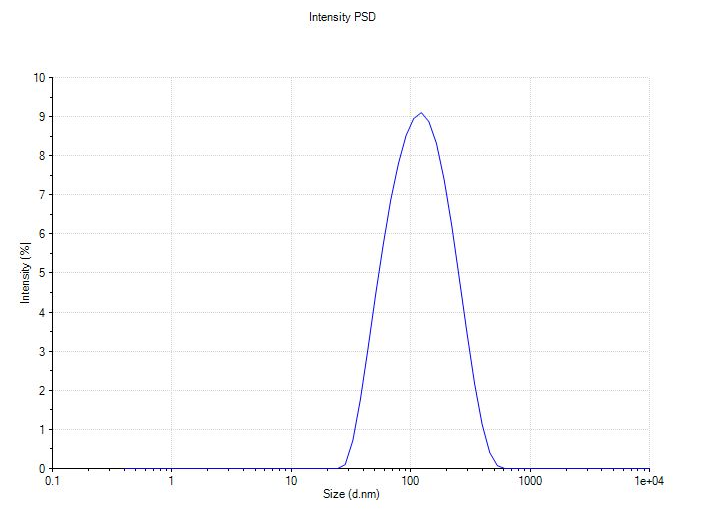

Supplement: Supplementary file 1 — Supplementary material 1. [file 41182_2024_636_MOESM1_ESM.docx]

**Supplementary file (S2).** Zeta potential distribution (mV)


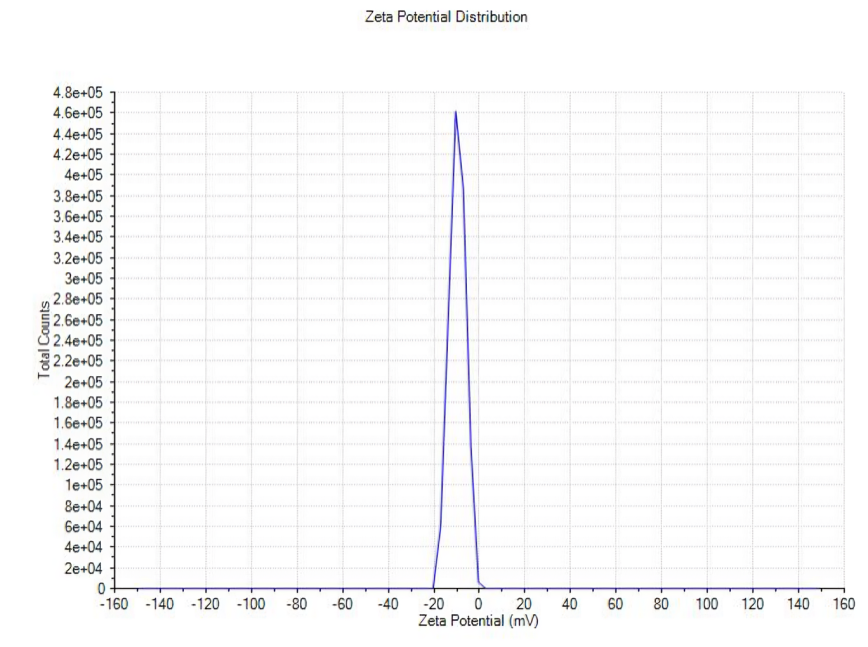

Supplement: Supplementary file 2 — Supplementary material 2. [file 41182_2024_636_MOESM2_ESM.docx]
